# International border malaria transmission in the Ethiopian district of Lare, Gambella region: implications for malaria spread into South Sudan

**DOI:** 10.1186/s12936-023-04479-5

**Published:** 2023-02-22

**Authors:** Werissaw Haileselassie, Abebe Ejigu, Tesfahun Alemu, Sale Workneh, Mizan Habtemichael, Randy E. David, Kidane Lelisa, Wakgari Deressa, Guiyun Yan, Daniel M. Parker, Behailu Taye

**Affiliations:** 1 School of Public Health, College of Health Sciences, Addis Ababa University, Addis Ababa, Ethiopia; 2 School of Pharmacy, College of Health Sciences, Addis Ababa University, Addis Ababa, Ethiopia; 3 Gambella Regional Meteorology Service Center, Gambella, Ethiopia; 4 School of Medicine, Wayne State University, Detroit, MI USA; 5 Department of Biology, Faculty of Natural and Computational Science, Dilla University, Dilla, Ethiopia; 6 Program in Public Health, College of Health Sciences, University of California at Irvine, Irvine, CA 92697 USA; 7 Department of Biology, Faculty of Natural and Computational Science, Mettu University, Mettu, Ethiopia

**Keywords:** Malaria, Lare, Western Ethiopia, South Sudan, Rural health, Community health

## Abstract

**Background:**

Despite notable progress in the control and prevention of malaria in the Horn of Africa, the disease continues to cause significant morbidity and mortality in various regions of Ethiopia, and elsewhere in the region. The transmission of malaria is affected by genetic, sociocultural, and ecological factors. Lare is an Ethiopian district adjacent to the Ethio-South Sudan border, in Gambella region. The region currently has the highest prevalence of malaria in Ethiopia. This study assesses the burden and spatiotemporal patterns of disease transmission, including the effect of climatic factors on the occurrence of malaria, across an international border crossing. This understanding can assist in crafting informed programmatic and policy decisions for interventions.

**Methods:**

This study was conducted in Lare district, Southwest Ethiopia, a temperate zone. A retrospective descriptive analysis was conducted using clinical service data collected between 2011 and 2021 from the 9 health facilities of the district. Both clinically diagnosed patients and those identified using microscopy and rapid diagnostic testing (RDT) were included in the study. Additionally, climate data was incorporated into analyses. Examples of analyses include malaria burden, positivity rate, incidence, species frequency, and an ANOVA to assess inter-annual case number and meteorological factor variation.

**Results:**

Between 2011 and 2021, a total of 96,616 suspected malaria cases were tested by microscopy or RDT, and 39,428 (40.8%) of these cases were reported as positive. There were 1276 patients admitted with 22 deaths recorded. There were further more significant fluctuations in positivity rates across years, the highest being 74.5% in 2021. Incidence varied from 18.0% in 2011 to 151.6% in 2016. The malaria parasite species most detected was *Plasmodium falciparum*, followed by a smaller proportion of *Plasmodium vivax*. The greatest proportions of *P. falciparum* cases were observed in 2018 and 2019, at 97.4% and 97.0% prevalence, respectively. There was significant seasonal variation in case number, the highest observed in July through September of each year. Climatic conditions of annual rainfall, temperature and humidity favored the increment of malaria cases from June until October.

**Conclusion:**

The study shows that the burden, i.e. morbidity and mortality (with fluctuating patterns) of malaria are still significant public health problems and can pose serious consequences in the district. This has implication for cross-border malaria transmission risk due to considerable border crossings. The predominant cause of the disease is *P. falciparum*, which causes severe complications in patients. The district has to prepare to deal with such complications for better patient care and outcomes.

## Background

Malaria is an infectious disease prevalent in the global tropics and sub-tropics, caused by *Plasmodium* Protozoa, and transmitted by *Anopheles* mosquitoes [1]. According to the 2022 World Malaria Report of the World Health Organization (WHO), there were 247 million cases in 2021 across eighty-four malaria endemic countries [2]. Although there was a consistent reduction of cases between 2000 and 2015, the number of cases has been increasing since 2016 largely attributed to disruptions caused by COVID-19 pandemic [3]. The incidence of malaria, defined as cases per 1000 population, in the world was 82.3 and 57.2 in 2000, and 2019, respectively, which shows an apparent reduction. However, it went up to 59.2 in 2020 without any change through 2021. Similarly, the global malaria mortality rate (deaths per 100, 000 population at risk) showed a remarkable reduction between 2000 and 2019 (30.1% in 2000, and 14% in 2019). The mortality was reported to be 14.8 in 2021 [2], a slight increase from 2019.

The Global Technical Strategy (GTS) for malaria 2016–2030 was adopted by the WHO in May 2015.The GTS promotes a community-based approach to accessing prevention, diagnosis, and treatment methods, as part of Universal Health Coverage (UHC) [4], which is meant to provide the full range of health services to individuals and communities through minimizing their financial hardships and improving geographic access [5].

In the past two decades, there has been a noteworthy reduction in malaria morbidity and mortality in Ethiopia, due in part to international investment in interventions [6–8], and particularly after the implementation of the national health strategic plan-Health Sector Transformation Plan (HSTP) I (2015–2020). Malaria incidence declined from 5.2 million in 2015 to less than 1 million in 2019. Deaths due to malaria decreased from 3.6 to 0.3 per 100,000 population at risk [6, 10]. However, Ethiopia still constitutes 1.7% of the global malaria cases. In 2021, there were 2, 783, 816 cases and 8,041 deaths [2]. It is estimated that approximately 60% of the population lives in at-risk areas [6, 9]. Malaria is one of the primary causes of hospitalization and death [9, 10], and remains as a significant public health problem.

In 2017, the Ministry of Health in Ethiopia introduced a sub-national malaria elimination programme targeting specific at-risk 238 districts, with a goal of achieving nationwide elimination by 2030 [8]. To augment the program, the second phase of the HSTP (2020 to 2025) is under implementation with the primary objectives of strengthening malaria surveillance and epidemic response, promoting sub-national malaria elimination, facilitating and enhancing microscopic examination and RDT, strengthening vector control, and impeding transmission among migrant workers [11].

Despite notable achievements in both morbidity and mortality due to malaria and intensified interventions at the moment in Ethiopia, “high level resistance of the vector to insecticides, suboptimal usage of interventions by target communities, complacency in maintaining the momentum, delay in implementing the national case manager guidelines, and shortage of complete and timely data for evidence-based decision making” are reported as the chief ongoing challenges [11].

Gambella has the highest prevalence (6%) of malaria of all the 11 regions and 2 administrative cities in the country [12]. All the 11districts within Gambella are malaria endemic. Lare is one of these malarious districts, for which scant information about regional epidemiology is available although all the people residing in it are at risk of acquiring malaria. More importantly, the porousness of the Ethio-South Sudan border coupled with high social cohesion between the border communities has contributed to considerable international cross-border mobility. Thus, understanding the malaria situation in the area could be of interest for informed programmatic and policy decisions to realize malaria related goals at different levels. Specifically, for efficient and effective resource allocation, spatiotemporal resolution of the burden of malaria is quite important.

## Methods

### Study setting

**Fig. 1:**
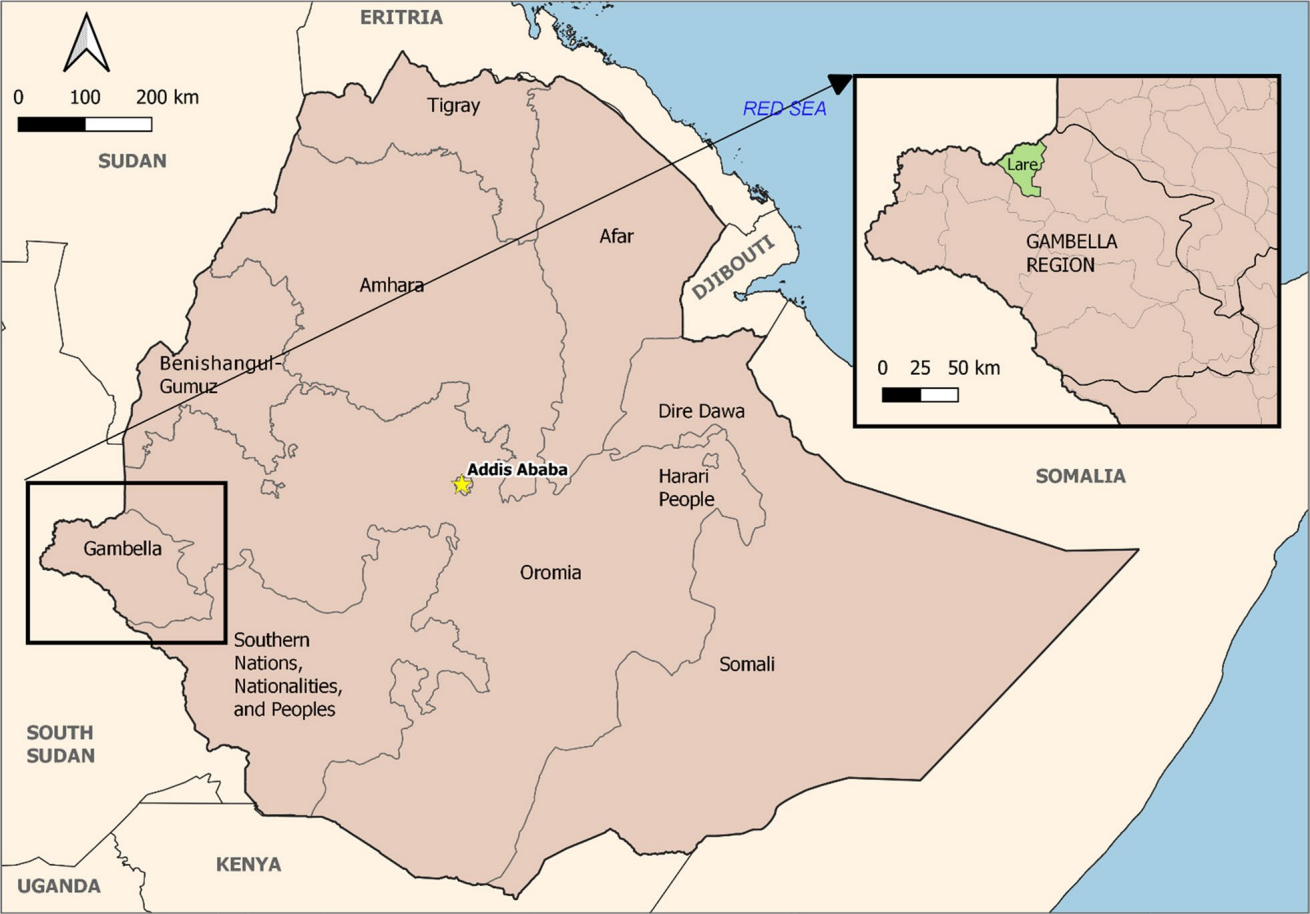
Map of Lare district, Gambella region, Ethiopia

The study area, Lare district, is situated in Nuer zone, within the Gambella region of southwestern Ethiopia. It shares a border with Anuak zone to the south and southeast. The Baro and Jikawo rivers separate Lare from Jikawo district to the east and northeast. South Sudan borders Lare district to the north and west. Lare is located at 33.95´N and 8.33´E with an elevation ranging from 410 to 430 m. Hot, humid weather conditions are typical in Lare, and terrain consists of largely wetlands and savannah. Lare is home to an estimated 54,070 residents. Two health centres and seven health posts currently provide clinical services to the community. Pastoralism is the primary provisioning activity in the district(Fig 1) [13, 14].

### Study design

A retrospective descriptive analysis was conducted using routine clinical service data collected from health facilities in Lare. Health service records from 2011 through 2021 were reviewed to extract data on malaria morbidity and mortality. Climate data for the district were obtained from the Gambella Meteorology Service Center.

### Data collection

Retrospective clinical service data related to malaria between 2011 and 2021were collected by health professionals from all the nine health facilities (2 health centres and 7 health posts) in the district. A health post is the lowest health facility at the primary level of the health care system that provides mainly essential promotive and preventive services and limited curative services at the community level below a district [15]. A health centre is a health facility at primary level of the healthcare system which provides promotive, preventive, curative and rehabilitative outpatient care including basic laboratory and pharmacy services with the capacity of 10 beds for emergency and delivery services [16]. Data recording was both in the morning and evening shifts. Hence, there was no jump in the data collection. In Ethiopia malaria is a weekly reportable disease. Thus, data was extracted from the weekly reports of the health facilities in the district.

Both clinically diagnosed malaria cases and those identified using microscopy and/or Rapid Diagnostic Test (RDT) were included in this study. Climate data on minimum, maximum and mean temperature, total rainfall, and relative humidity of the district were obtained from the Gambella region Meteorology Service Centre.

### Data analysis

Data was entered into a database and “cleaned” for completeness and accuracy on R [17]. It was then analyzed using R and SPSS version 20 (SPSS Inc., Chicago, II).

#### *Malaria incidence* was calculated as

Total number of confirmed malaria cases divided by the total population at risk multiplied by one thousand.

#### Malaria test positivity was calculated as

Number of slides/RDT positive for malaria divided by total number of slides/RDT performed for malaria multiplied by one hundred.

#### The percentage of Plasmodium falciparum cases was calculated as

Number of confirmed *P. falciparum* malaria cases divided by total number of confirmed malaria cases multiplied by one hundred.

An ANOVA was performed to assess variability between inter-annual case number and meteorological factors. Annual species composition was calculated for malaria parasites. Seasonality by total malaria cases was also determined. A Simple Seasonal Model was performed to forecast future annual malaria cases.

### Ethical considerations

This study was approved by the Institutional Review Board of the College of Health Sciences, Addis Ababa University (AAUMF 03-008). Furthermore, a letter of permission was attained from local health authorities to access to health facility records. Confidentiality of protected health information was maintained using physical and password-protected digital restrictions. The data were paper-based and aggregated or compiled by the facilities as their routine report and, consequently, there was not any possibility to identify any individual.

## Results

### Trends in malaria morbidity and mortality in Lare district

**Fig. 2:**
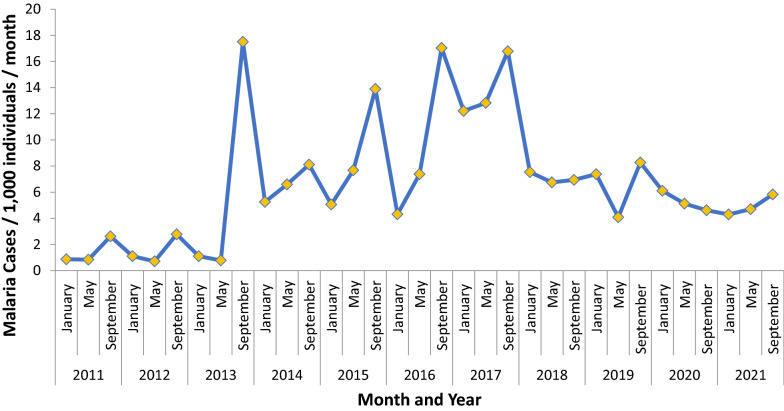
Trends of malaria incidence in Lare district, Ethiopia, 2011–2021

Eleven years (2011–2021) of malaria case incidences from Lare district were analysed from data of individuals with symptoms who visited the health facilities for healthcare services. According to Fig. 2, 2016 had the highest malaria case incidence rate at 17.49 per 1000. There were noteworthy peaks in the month of September in years 2013, 2015, 2016, and 2017. There was a substantially lower number of cases from 2011 to 2012 and 2018 onward.

**Fig. 3:**
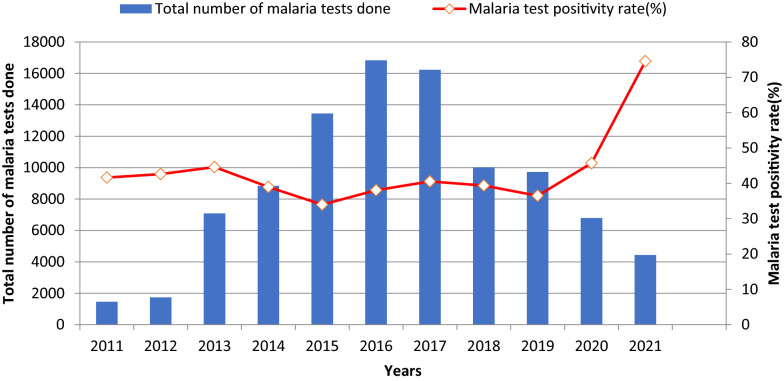
Completed tests and test positivity rate in Lare district, Ethiopia, 2011–2021

Between 2011 and 2021, a total of 96,616 individuals were tested for malaria by either direct microscopy or RDT, and 39,428 (40.8%) were found to be positive (Fig. 3). The greatest annual test positivity rate was observed in 2021 (74.5%), followed by 2020 (45.7%). There was significant variability in annual number of tests performed with a relatively steady increase in testing from 2011 (1458 tests) to 2016 (when testing peaked at 16,840), followed by a drop off between 2016 and 2021 4432 tests).

**Fig. 4:**
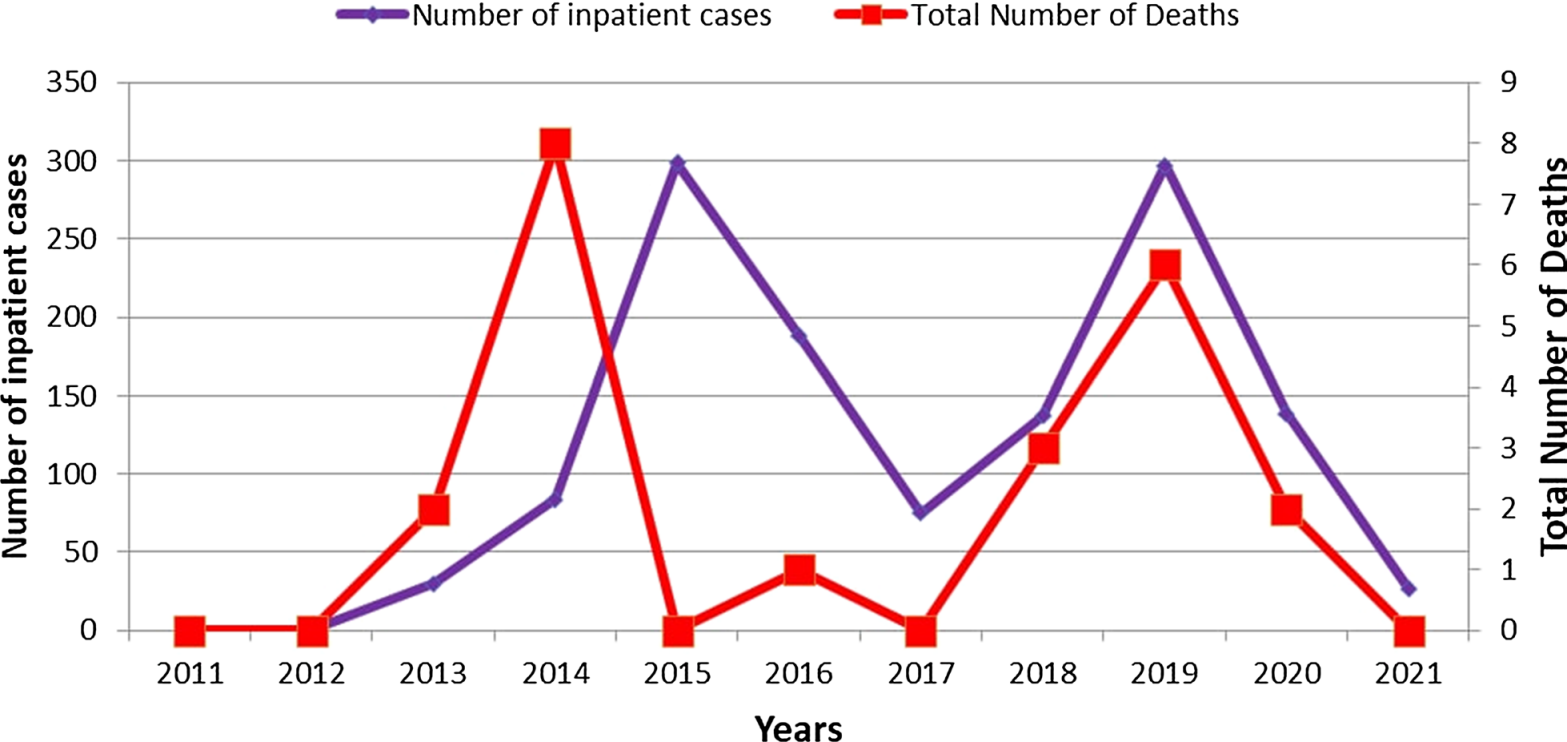
Malaria inpatients and deaths in Lare district, Ethiopia, 2011–2021

Between 2011 and 2021, a total of 1276 patients were admitted to one of the 9 health facility in the district for treatment of malaria (Fig. 4). The number of inpatients and deaths fluctuated drastically on an annual basis during this 11-year period. In 2014, only 84 inpatients were treated. This increased to 299 by the following year (2015). In 2012, there were 0 deaths. Two years later, in 2014, there were 8 deaths reported. Overall, between 2011 and 2021, 22 individuals died of malaria.

**Fig. 5:**
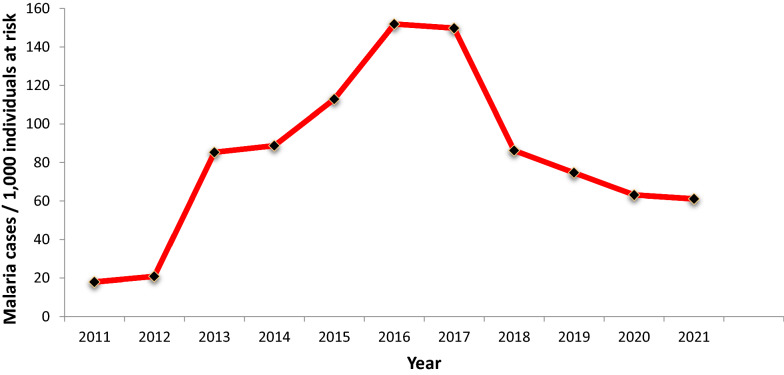
Burden of malaria over the past eleven years in Lare district, Gambella, Ethiopia, 2011–2021

Figure 5 depicts the incidence of malaria per 1000 individuals at risk for malaria, from 2011 to 2021. Incidence in 2011 and 2012 was relatively consistent, at 18.0 per 1000 and 20.9 per 1000, respectively. Incidence increased to 85.2 per 1000 in 2013, then increased sharply, reaching its peak in 2016, at 151.6 per 1000. From 2018 to 2021, incidence decreased from 86.19 per 1000 to 61.12 per 1000.

### Malaria morbidity by species

**Fig. 6:**
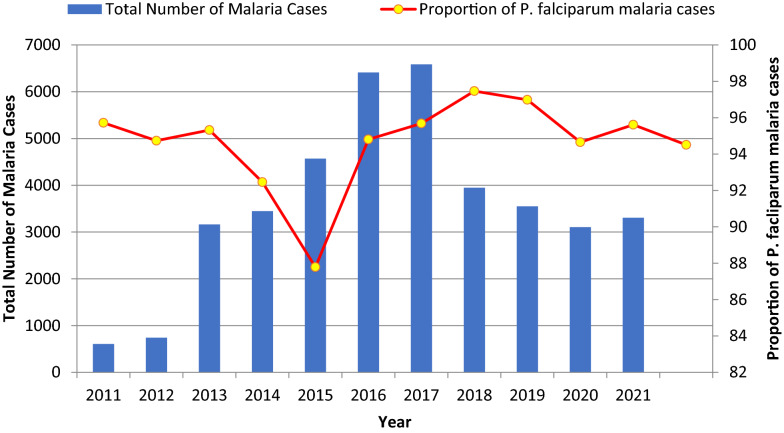
Total malaria cases and proportion of P_f_ cases in Lare district, Ethiopia, 2011–2021

As illustrated in Fig. 6, *P. falciparum* was the predominantly reported species throughout the study period, accounting for 94.5% of malaria cases. *Plasmodium vivax* constituted 5.35% of cases. The greatest proportion of *P. falciparum* cases were observed in 2018, at 97.4%. *P. falciparum* showed a relatively lower prevalence in 2015, at 87.8%.

**Fig. 7:**
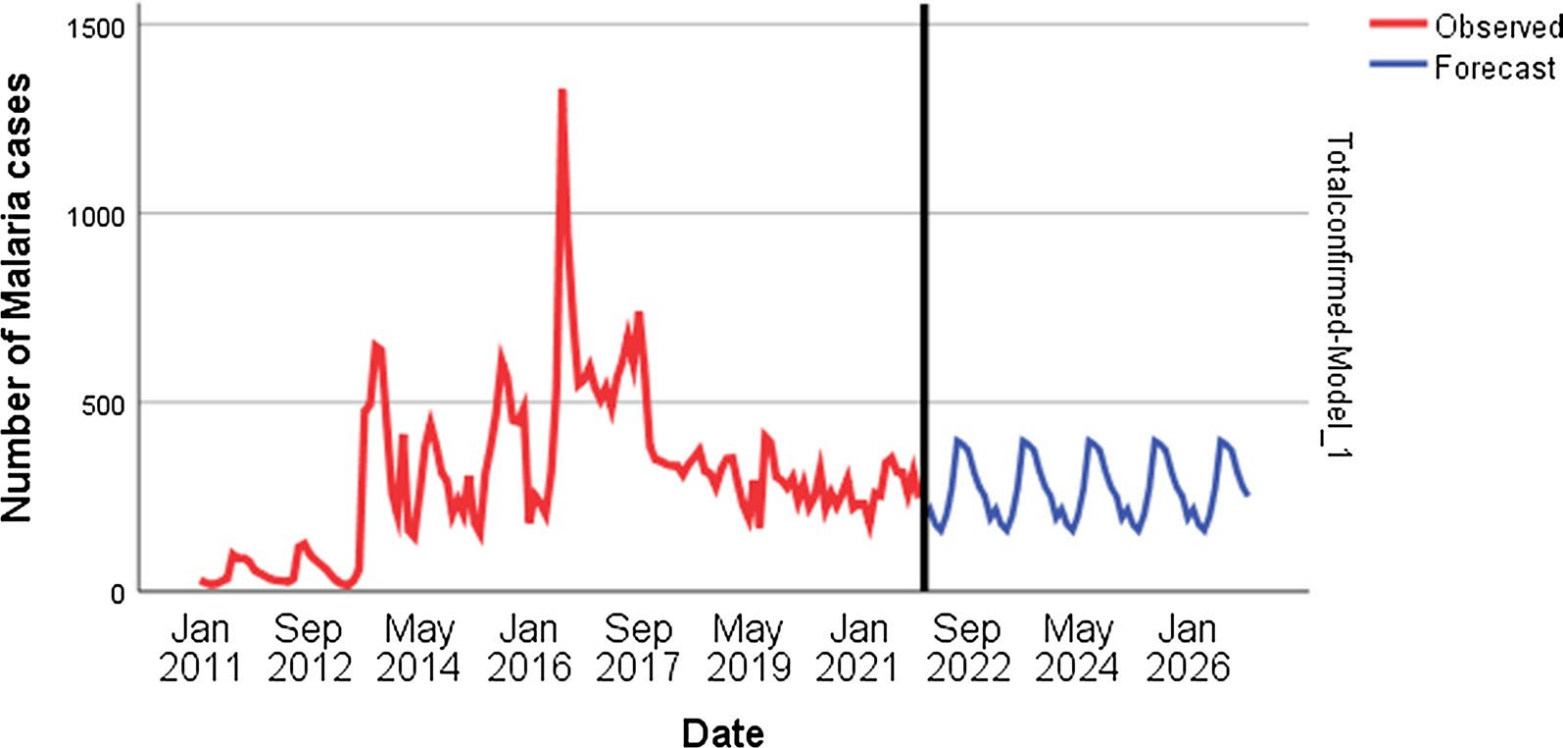
Five-year forecast of malaria cases in Lare district, Ethiopia, 2022

### Seasonal prevalence of malaria, and forecasting

Malaria cases were reported in all months of the 11-year study period, with notable seasonal variation, which translates into sub perennial transmission. The greatest number of malaria cases were observed from July to September. Cases slightly decreased from October to December. The lowest number of malaria cases was reported from March to May. A significant effect of seasonality on total malaria cases was observed (F_3,8_ = 9.592, P = 0.005). The inter-annual total case count difference was also significant (F_109,22_ = 2.737, P < 0.004). Though malaria showed reduction over the past few years, the forecast showed that it will maintain its current level of occurrence (Fig 7).

### Trends in total malaria cases and meteorological factors

**Fig. 8:**
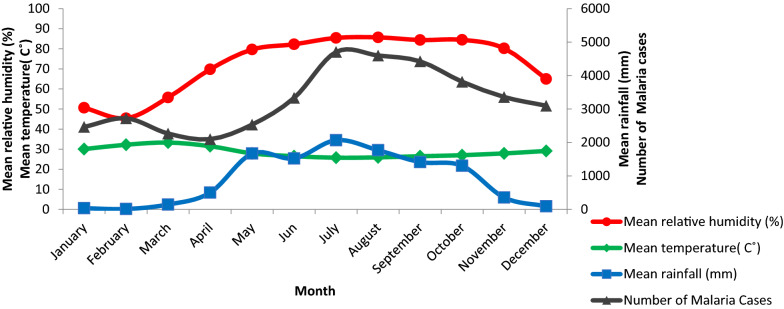
Malaria cases and monthly meteorological factors in Lare district, Ethiopia, 2011–2021

As shown in Fig. 8, across the 11-year study period, climatic conditions in July and August were most favorable for malaria cases. Visually, it appears that an increase in cases was strongly associated with rainfall, and relative humidity. In Lare district, mean rainfall was 2069.90 mm and relative humidity was 85.43% in July. There was a significant difference between interannual relative humidity in the district (F_206,69_ = 1.721, P = 0.005).

## Discussion

Ethiopia has registered significant progress in reducing the burden of malaria over the past two decades [18]. This is likely the result of improved implementation of high impact interventions [1], including prompt treatment of cases using artemisinin-based combination therapy [3], prevention and control using intermittent preventive therapy [10] vector control methods including insecticide-treated bed nets and indoor residual spray, and [9] the effective distribution of diagnostic tests [19, 20]. Despite a noteworthy decline in malaria cases and deaths, malaria remains one of the leading causes of morbidity and mortality in Ethiopia [21, 22].

This study indicated that the number of confirmed malaria cases within the eleven-year period was 39,428. Although the test intensity had shown reduction over the past few years (2019–2021), malaria positivity rate increased during the same time period. The reduction in test intensity might be due to service disruption following COVID 19 pandemic whereas the increased confirmation of malaria could be due to improved diagnostic service quality through the external quality assurance mechanism (EQA) and supply of laboratory diagnostic tools and reporting, rather than a true rise in malaria prevalence [23]. The 11-year test positivity rate of 38.5% was comparable with other studies recently conducted in Ethiopia such as in Kola Diba district (39.6%) [24], Omo zone (39.6%) [25] and Woreta town (32.6%) [26]. The test positivity rate of the present study is higher than study findings in Kombolcha (7.52%) [27], Bahir Dar (5%) [28] towns, Arsi Negelle district (11.40%) [29], and Northern Shewa (8.4%) [30] and Wellega (20.7%) [31] zones of Ethiopia. A reduction in malaria cases may be related to the scaling up of malaria prevention outreach and education that increased awareness in the community towards the application of different insecticides and repellents, and use of ITNs [32]. It is important to note that malaria infection in Ethiopia is highly variable and unstable due to drastic differences in environmental, climatic, and sociocultural conditions, as well as variability in intervention investments.

The dominant species of malaria parasite during the study period was *P. falciparum*, which accounted for 94.7% of cases. This finding is congruent with malaria species distribution in several parts of Ethiopia, including Abobo district (90%) [33] in Gambella region, and Bale zone (81.5%) [34]. The study area exhibits a higher proportion of *P. falciparum* than the corresponding national figure (81.6%) [35]. The dominance of *P. falciparum* indicates the increased likelihood of severe cases in the study area, and underscores the importance of sub-national tailoring of malaria prevention and intervention. Unlike other areas of Ethiopia, malaria cases were reported year-round in Lare district. The highest number of cases and detection rate of malaria were observed between June and August, a similar finding to studies conducted in Oromia region, Wolaita zone, Northwest Tigray and Northern Shewa [30, 32, 36, 37].

There are significant movement of people in and out of Gambella Region during months of high malaria transmission [38]. This can lead to the movement of parasites within and outside of the region. It in turn contributes for persistence of transmission in areas where there are competent human malaria vectors. This will frustrate malaria elimination efforts in the region. Moreover, the local movement of folks during times of the day when mosquito vectors are active was also significant. This will lead to residual transmission, even if there is widespread coverage and use of bed nets in the area [38]. Besides, noticeable international border crossings along the Lare route into South Sudan (personal communication and observation) facilitate malaria spread through parasite transportation thereby challenging malaria control and elimination efforts in the Horn of Africa.

It is important to note that, in Lare district, as elsewhere in Ethiopia, peak malaria transmission often coincides with the planting and harvesting seasons. This highlights the importance of outreach to rural agrarian communities and migrant agricultural workers.

The mean annual incidence of malaria during the study period was 85.5 cases/1,000 population at risk. The result of the study is higher than the national annual incidence rates of malaria in 2014, 2015, and 2016, which were 54.2, 45.7, and 40.3 per 1,000 population at risk [34]. Nonetheless, as of 2017, Ethiopia still accounted for approximately 6% of global malaria cases and 12% of global *P. vivax* cases and deaths [39].

In this study, secondary data from health facilities were used to determine the trends in malaria morbidity and mortality. The registered data lacks important variables such as age in case data and age and sex in death record. Therefore, one limitation of this study was in disaggregating malaria surveillance data by age, sex, and age*sex. Moreover, as this study used secondary data to analyse the malaria situation in a district, it might have some limitations related to this data source.

## Conclusion

The study shows that the burden i.e. morbidity and mortality (although fluctuating patterns) of malaria are still significant public health problems and can pose serious consequences in Lare district. This has implication for cross-border malaria transmission risk due to considerable border crossings into South Sudan. Moreover, *P. falciparum* is the dominant cause of malaria, increasing the likelihood of developing severe complications from malaria, compared to other types. As usual, the pattern of the occurrence of cases is accompanied by concomitant seasonal and meteorological changes or variations. However, cases are reported year-round. The in and out movement of people in the district. In addition, similar patterns of disease burdens of prevalence and incidence are likely in the border districts of South Sudan. It is recommended that the district needs to have the necessary preparation and facilities to diagnose and treat severe malaria complications during the peak seasons of the disease. Moreover, cross-border coordination on malaria diagnosis and treatment could be considered to mitigate the impact of the disease. Planning and implementation of joint vector control and case management activities are very important for countries that are already embarked on malaria elimination. Moreover, we also suggest sharing of epidemiological data, joint resource mobilization and sharing, and harmonizing capacity building for overall joint implementation along border regions.

## Data Availability

Data supporting the result are included within the article.

## Abbreviations

GTS: The global technical strategy
HSTP: Health sector transformation plan
MOH: Ministry of health
RDT: Rapid diagnostic testing
UHC: Universal health coverage
WHO: World health organization
